# Maternal smoking during pregnancy and autism: using causal inference methods in a birth cohort study

**DOI:** 10.1038/s41398-018-0313-5

**Published:** 2018-11-29

**Authors:** Doretta Caramaschi, Amy E. Taylor, Rebecca C. Richmond, Karoline Alexandra Havdahl, Jean Golding, Caroline L. Relton, Marcus R. Munafò, George Davey Smith, Dheeraj Rai

**Affiliations:** 1 Medical Research Council Integrative Epidemiology Unit, University of Bristol, Bristol, UK; 2 Population Health Sciences, Bristol Medical School, University of Bristol, Bristol, UK; 3 Centre for Child and Adolescent Health, Bristol Medical School, University of Bristol, Bristol, UK; 4 School of Experimental Psychology, University of Bristol, Bristol, UK; 5 National Institute for Health Research Bristol Biomedical Research Centre, University Hospitals Bristol NHS Foundation Trust and the University of Bristol, Bristol, UK; 6 Centre for Academic Mental Health, Bristol Medical School, University of Bristol, Bristol, UK; 7 Avon and Wiltshire Partnership NHS Mental Health Trust, Bristol, UK

## Abstract

An association between maternal smoking in pregnancy and autism may be biologically plausible, but the evidence to date is inconsistent. We aimed to investigate the causal relationship between maternal smoking during pregnancy and offspring autism using conventional analysis and causal inference methods. In the Avon Longitudinal Study of Parents and Children we investigated the association of maternal smoking during pregnancy (exposure) with offspring autism spectrum disorder (ASD) or possible ASD diagnosis (*n* = 11,946) and high scores on four autism-related traits (outcomes) (*n* = 7402–9152). Maternal smoking was self-reported and also measured using an epigenetic score (*n* = 866–964). Partner’s smoking was used as a negative control for intrauterine exposure (*n* = 6616–10,995). Mendelian randomisation (*n* = 1002–2037) was carried out using a genetic variant at the *CHRNA3* locus in maternal DNA as a proxy for heaviness of smoking. In observational analysis, we observed an association between smoking during pregnancy and impairments in social communication [OR = 1.56, 95% CI = 1.29, 1.87] and repetitive behaviours, but multivariable adjustment suggested evidence for confounding. There was weaker evidence of such association for the other traits or a diagnosis of autism. The magnitude of association for partner’s smoking with impairments in social communication was similar [OR = 1.56, 95% CI = 1.30, 1.87] suggesting potential for shared confounding. There was weak evidence for an association of the epigenetic score or genetic variation at *CHRNA3* with ASD or any of the autism-related traits. In conclusion, using several analytic methods, we did not find enough evidence to support a causal association between maternal smoking during pregnancy and offspring autism or related traits.

## Introduction

Autism spectrum disorders (ASDs) are complex neurodevelopmental disorders characterised by persistent deficits in social communication and social interactions and by restricted, repetitive patterns of behaviours, interests or activities. There is increasing evidence to suggest that environmental factors around birth may play a role in the aetiology of ASD. Maternal smoking during pregnancy has been implicated in the aetiology of autism^1^. An increasing body of literature has reported a range of adverse neuro-behavioural outcomes consequent to maternal smoking including the risk of other neurodevelopmental disorders, particularly externalising problems such as attention-deficit hyperactivity disorder (ADHD)^2,3^. Smoking during pregnancy is also known to be associated with neonatal complications that could be on the causal pathway to later neurodevelopmental problems^1^. Prenatal exposure to smoking also leads to epigenetic modifications that are apparent in blood and that persist throughout childhood and adolescence and might impair neurodevelopment^4^. Several population-based studies investigating the association between maternal smoking during pregnancy and offspring ASD have returned inconsistent results^1,5–15^ and meta-analyses of these studies did not support an association^16–18^. However, a recent meta-analysis suggests that taking other factors into account such as paternal smoking and second-hand smoking could account for the heterogeneity between the different studies^19^. Altogether these studies remain inconclusive.

One possible reason behind the inconsistent findings is that smoking in pregnancy is not uniformly distributed in the population and is highly socially patterned. Consequently, any observed associations could be confounded by socioeconomic factors^11^. However, despite attenuation of associations in some studies^6,11^, others reported ‘positive’ associations of maternal smoking with ASD, which persisted after adjustment for socioeconomic factors^8,13,14^, and some studies did not find any primary association^5,6,9,12^. Even after adjusting for measured variables, residual or unmeasured confounding is a potentially important issue in observational studies^20^. Unmeasured confounders could potentially include maternal factors that relate to their mood or stress levels during pregnancy, as these have been linked with both smoking behaviour and ASD^21,22^. However, previous studies that found an effect of maternal smoking on ASD did not adjust specifically for mood disorders during pregnancy.

Among various approaches to address confounding, Mendelian randomisation (MR) has been used to investigate causality where observational associations have been previously found. In a MR framework, a genetic variant is used as a proxy for the exposure of interest and, since genetic variants are randomly allocated at conception, they are not liable to confounding^23^. Previous large-scale genetic population studies have identified genetic variants that are strongly associated with smoking behaviour. The single-nucleotide polymorphism (SNP) rs1051730:G > A in the *CHRNA5-CHRNA3-CHRNAB4* gene cluster coding for nicotinic receptor subunit proteins^24,25^ is strongly associated with heaviness of smoking in smokers (i.e. number of cigarettes smoked per day). MR studies using this gene variant for smoking during pregnancy have added evidence to determine whether it has consequences for prenatal depression and birth weight^26–28^.

It has also been suggested that smoking is often misreported, especially during pregnancy, where the behaviour is considered socially undesirable^29^. Biomarkers may be preferable to provide a more objective measure of smoking exposure, and recent epigenetic scores derived from DNA methylation data have been proposed to be used in place of self-reported smoking behaviour^30^.

Another way to determine whether maternal smoking exposure has a causal effect on risk of ASD is to include a negative control exposure in the analysis^31^. Partner’s smoking during pregnancy is as socially patterned as maternal smoking therefore its influence on the child’s neurodevelopment may arise from sharing the same socioeconomic confounders with maternal smoking. However, maternal smoking can affect the risk of developing ASD directly via intrauterine mechanisms, assuming the effects of passive smoking from exposure to partner smoking are minimal. For instance, cotinine levels in pregnant women were found to be mostly associated with their own smoking status, rather than with their partner’s^32^. Partner’s smoking acts therefore as a negative control for the main exposure and we would expect its association with ASD to be weaker than that of maternal smoking with ASD if in utero smoke exposure was causal.

Furthermore, there is evidence to suggest that impairments in various domains used to describe ASD may have varying aetiologies^33^. Studies investigating component traits of ASD may therefore be useful in the identification of ASD risk factors as maternal smoking might be differentially harmful for the different symptoms of ASD. ASD-related traits can also present in children without ASD diagnosis and therefore population-based studies that consider autistic traits are informative as the cut-off for diagnosis is often arbitrary and almost all traits occur in a continuum of severity^34^.

In this study we investigated the causal effect of maternal smoking during pregnancy on children’s ASD and ASD traits using three approaches. First, we used the traditional observational analysis (self-reported smoking and epigenetic score) adjusted for potential confounders. We then investigated the effect of partner’s smoking as a negative control. Finally, we performed exploratory MR using a maternal genetic variant as proxy for smoking heaviness.

## Materials and methods

### Study population

The Avon Longitudinal Study of Parents and Children (ALSPAC) is a birth cohort based in Avon, UK^35,36^. Data were collected via self-completed questionnaires administered to the mothers at four time points during pregnancy, to the partners at two time points during pregnancy and to the mothers and their partners (mainly fathers) at regular intervals following birth. Since the age of 7 years the children were also administered questionnaires. Ethical approval for this study was obtained from the ALSPAC Ethics and Law Committee and the Local Research Ethics Committees.

The study website contains details of all the data that are available through a fully searchable data dictionary: http://www.bris.ac.uk/alspac/researchers/data-access/data-dictionary.

### Exposure: prenatal smoking

Maternal smoking status during pregnancy was obtained from the questionnaires administered to the mothers at 8, 18–32 weeks of gestation and just after birth. These data were combined into a dichotomous variable for any reported smoking vs. none.

Maternal smoking was also assessed using a smoking epigenetic score that was created for each of the ALSPAC mother–child pair with DNA methylation measured as part of the Accessible Resource for Integrated Epigenomic Studies^37^. Details of data generation can be found in the Supplementary Material. From the beta values of mother’s DNA methylation data collected antenatally, methylation scores were calculated using three different methods: (i) the Elliott score was calculated using the procedure reported in Elliott et al.^30^, with summary statistics taken from Zeilinger et al.^38^; (ii) the Joehanes score was calculated as a weighted additive score using the 2622 sites at *p* < 10^−8^ from Supplementary Table 2 in Joehanes et al.^39^; (iii) the Joehanes-PC-adjusted score was calculated as the Joehanes score except the beta values were adjusted for 10 principal components that derived from the most variable 10,000 CpG sites prior to the score calculation. From the beta values of cord blood methylation, two methylation scores were computed: (i) the Joubert score was calculated using as a weighted additive score using 568 sites from Supplementary Table 3 in Joubert et al.^40^; (ii) the Joubert-PC-adjusted score was calculated as the Joubert score except the beta values were adjusted for 10 principal components that derived from the most variable 10,000 CpG sites prior to score calculation. The five scores generated were then compared in terms of predicting pregnancy smoking and the best predictor based on the pseudo-*R*^2^ was chosen for the main analysis.

Partner’s smoking status during pregnancy was obtained from questionnaires administered to the mothers at 18 weeks of gestation regarding their partners’ current smoking status and to the partners asking about their own smoking habits during the pregnancy period. A categorical variable with current smokers vs. non-smokers (either never smoked or stopped smoking) was created.

### Outcome variables

#### Autism spectrum disorder

Children with ASD within ALSPAC were identified using a multisource approach: (a) a review of all children given a statement for special educational provision in the Avon area to identify children with a diagnosis of ASD concordant with the International Classification of Disease (10th Edition) (ICD-10) criteria^41^; (b) the mother’s answer to the(question “Have you ever been told that your child has autism, Asperger’s syndrome or ASD?” when the child was 9 years old; (c) classification by the educational system as requiring special needs because of an ASD by age 16; (d) text responses to any questions on child’s diagnoses in questionnaires administered when the child was between 6 months and 11 years; and (e) ad hoc letters from parents to the Study Director^42^. Using these sources, 212 offsprings with reported or possible ASD diagnosis were identified and 174 of these had information collected prospectively during pregnancy. This method for defining ASD cases in ALSPAC has been previously validated^42^.

#### ASD-related traits

ASD traits used as outcomes in this study were four measures derived from parental questionnaires in ALSPAC, which were previously reported to optimally predict ICD-10 ASD diagnosis out of 93 measures related to autistic features collected in ALSPAC by age 11 years^43^: (1) a social communication impairment score derived by administering the Social and Communication Disorders Checklist (SCDC) questionnaire to the mothers when the children were 7.5 years of age^44^; (2) the Coherence subscale of the Children’s Communication Checklist (CCC) that was administered at 9.5 years of age^45^ as a measure of pragmatic language skills; (3) a Repetitive Behaviour score was derived from the answer to four questions sent to the mothers at 69 months regarding how often the child repeatedly rocks the head or body for no reason has a tic or a twitch, has other unusual behaviour and whether they stumble, get stuck on words or repeat them many times^46^; and (4) the sociability subscale of the Emotionality Activity and Sociability (EAS) temperament scale, which measured at 38 months to assess the tendency to affiliate and interact with others^47^. Since three of the scores considered were highly skewed we dichotomised all the scores into low- and high-risk groups. The SCDC was dichotomised using a cut-off of ≥9 for the high-risk group as this threshold was previously validated^48^. For pragmatic language ability coherence and EAS sociability, we reverted the scale and created a high-risk group for ASD of as close to 10% of the population as possible. For repetitive behaviour we also created a high-risk group for ASD of as close to 10% of the population as possible. We also used the previously published ASD factor mean score from a factor analysis of all the 93 traits that were potentially related to ASD as an outcome representing the broad autism phenotype^43^.

### Covariates

A number of variables considered potential confounders were included in the adjusted analyses. These were offspring sex and several maternal variables: age, parity, education, social class, financial difficulties and antenatal depression. Maternal age was derived from the date of birth that was recorded in a questionnaire administered at 8 weeks. Parity (number of previous pregnancies resulting in live births or stillbirths) was recorded in a questionnaire administered to the mother at 18 weeks of pregnancy and was dichotomised into ‘no previous children’ and ‘at least one child’. For maternal education the highest education level reached was reported in a questionnaire at 32 weeks of pregnancy and was dichotomised into possessing a University degree or not. Occupational class was derived from the maternal occupation stated in questionnaires administered at 32 weeks of pregnancy and collapsed into two categories representing ‘manual occupations’ and ‘non-manual occupations’ (according to the 1991 British Office of Population Census Surveys job codes). Financial difficulties were recorded as having difficulties affording things for the baby, accommodation, heating, clothing and food and combined into a score (0–15). The score was dichotomised with a cut-off of ≥9 representing the 10% of the sample with high financial difficulties. Maternal depression was ascertained using the Edinburgh Postnatal Depression Scale measured at 18 weeks of pregnancy, which is a score ranging from 0 to 30 with higher scores indicating more severe depressive symptoms^49^.

#### Genetic variants for MR

Within a MR framework, the number of alleles at common genetic variants that are robustly associated with smoking behaviour is used as the exposure variable instead of the exposure of interest, smoking behaviour. Evidence of an association between the genetic variants for smoking in the study mothers and children’s ASD or autism-related traits would indicate a causal link. The SNP rs1051730:G > A within the *CHRNA3* locus was chosen to proxy for exposure to tobacco smoke in the MR study because of its strong association with heaviness of smoking, rather than smoking initiation or cessation, which might be more prone to socioeconomic patterns. Maternal genotype (number of alleles) at rs1051730:G > A was extracted from the ALSPAC genetic database, which contains genome-wide data on ALSPAC mothers and children^35^. Details of generation of the genetic data are provided in the Supplemental Methods.

### Statistical analysis

The association between maternal smoking and ASD/autistic traits was assessed using logistic regression models with self-reported smoking and epigenetic smoking score as exposure variables and with ASD diagnosis or individual ASD traits as outcome variables. Linear regression models were run when the ASD factor mean score was used as outcome. Partner’s smoking was used as a negative control exposure variable and its association with child ASD and autistic traits was estimated. First, we estimated associations adjusted for sex of the child. In the fully adjusted model we adjusted for sex, maternal age at delivery, parity, maternal education, social class and financial difficulties as potential confounders. We also ran a separate model that included both maternal and partner’s smoking together as a means of mutual adjustment. Both the maternal and partner’s smoking analyses were further adjusted for mother depression symptoms to rule out confounding by maternal depression.

MR analysis was carried out by assessing the effect of smoking-associated maternal genotype at the *CHRNA3* locus (rs1051730:G > A) on ASD and autistic traits. First, the association between maternal genotype and heaviness of smoking was confirmed in the study sample using linear regression with the number of A alleles (0, 1 and 2) for each genotype as the exposure variable and heaviness of smoking during pregnancy (number of cigarettes per day) as outcome within each trimester. We performed this analysis by trimester because heaviness of smoking varied in each trimester. Then, the effect of maternal genotype on ASD and autistic traits was assessed in logistic regression models for each SNP with number of A alleles at rs1051730:G > A as exposure and ASD or autistic traits as binary outcomes. Linear regression was used when the factor mean score for ASD was the outcome. The MR analysis was carried out stratified by smoking during pregnancy since the genetic variant is an instrument for heaviness of smoking within smokers and across the whole pregnancy rather than by trimester as the outcome sample size would be considerably reduced.

The power analysis estimated that based on our samples sizes and on the summary statistics of the control groups in our study we had, at alpha = 0.05, 80% power to detect: in the observational analysis of maternal smoking and paternal smoking an odds ratio of at least 1.63 for ASD diagnosis and at least 1.2–1.3 for the ASD-related traits; for smoking methylation score an odds ratio of at least 1.45 for ASD diagnosis and at least 1.07 for ASD-related traits; for number of smoking alleles an odds ratio of at least 2.45 for ASD diagnosis and at least 1.45 for ASD-related traits.

All the analyses were performed using STATA 15.0 (StataCorp LLC, Texas, USA) and R 3.3.3 (https://www.r-project.org/).

## Results

The baseline characteristics of the study populations are reported in Table 1 and show consistency across the samples for the three different exposures considered (maternal smoking during pregnancy, maternal genotype and partner’s smoking during pregnancy).

**Table 1:** Baseline characteristics of the study populations in the ALSPAC sub-samples with data on maternal smoking, partner smoking and with genotype data

Variable	Maternal smoking sample (*N* ≤ 12,044)^a^	Partner smoking sample (*N* ≤ 11,116)^a^	Maternal genotype sample (*N* ≤ 8771)^a^
Sex (%male)	51.83	51.54	50.20
ASD (%cases)	1.24	1.18	1.14
SCDC (%high score)	7.88	7.56	7.70
CCC coherence (%low score)	10.34	9.79	9.72
Repetitive behaviour (%high score)	6.95	7.03	6.75
EAS sociability (%low score)	11.59	11.34	11.43
Maternal education (university degree)	13.19	13.51	13.80
Social class (%manual)	19.50	19.38	18.04
Parity (%multiparous)	56.21	54.43	54.08
Mothers smoking (%)	30.68	30.15	27.45
Partners smoking (%)	42.51	43.39	40.64
Financial difficulties (%)	9.93	9.26	8.95
Maternal age, mean (sd)	28.22 (4.89)	28.15 (4.88)	28.34 (4.76)
EPDS score, mean (sd)	6.92 (4.85)	6.89 (4.82)	6.75 (4.75)

*ASD* autism spectrum disorder, *SCDC* Social and Communication Disorders Checklist, *CCC* Children’s Communication Checklist, *EAS* Emotionality Activity and Sociability, *EPDS* Edinburgh Postnatal Depression Scale

^a^*N* varies according to completeness of data on baseline characteristics

The association of potential confounding factors with mothers’ and partner’s smoking status during pregnancy is shown in Supplemental Table S1. Compared to mothers who did not smoke during pregnancy, mothers who did smoke had more boys, were on average 3 years younger, more often employed in manual jobs, had lower education, reported more financial difficulties and had higher depression scores. Similar associations were seen with partner’s smoking status during pregnancy.

In sex-adjusted models maternal smoking was associated with higher risk of social communication impairment SCDC score, increased repetitive behaviour and an increased ASD risk predicted by the factor mean score. There was an apparent protective association with ASD diagnosis (Table 2). There was strong evidence of confounding and these associations shifted towards the null upon adjustment with socioeconomic covariates, partner’s smoking and maternal depression.

**Table 2:** Association of maternal smoking during pregnancy with child’s autism spectrum disorder and ASD traits without adjustment and with adjustment for socioeconomic status, partner’s smoking and maternal depression

Outcome	Model 1			Model 2			Model 3			Model 4		
	*N*	OR [95% CI]	*p*-Value	*N*	OR [95% CI]	*p*-Value	*N*	OR [95% CI]	*p*-Value	*N*	OR [95% CI]	*p*-Value
ASD	11,946	0.66 [0.45, 0.97]	0.033	8986	0.67 [0.40, 1.12]	0.124	7684	0.83 [0.46, 1.50]	0.543	7274	0.84 [0.46, 1.56]	0.208
SCDC score	7402	1.56 [1.29, 1.87]	<0.0005	6142	1.52 [1.22, 1.89]	<0.0005	5380	1.31 [1.02, 1.70]	0.038	5135	1.23 [0.94, 1.60]	0.130
CCC coherence (reverted)	7096	1.19 [0.99, 1.42]	0.063	5899	1.08 [0.87, 1.34]	0.481	5182	1.13 [0.88, 1.46]	0.326	4952	1.04 [0.80, 1.34]	0.780
Repetitive behaviour	7854	1.34 [1.09, 1.61]	0.004	6474	1.28 [1.01, 1.62]	0.043	5665	1.21 [0.93, 1.59]	0.161	5415	1.09 [0.82, 1.44]	0.549
EAS sociability (reverted)	9152	0.99 [0.85, 1.15]	0.891	7392	0.85 [0.71, 1.02]	0.089	6421	0.84 [0.67, 1.03]	0.097	6119	0.80 [0.64, 1.00]	0.051
	***N***	**Beta** **[95% CI]**	***p*** **-Value**	***N***	**Beta** **[95% CI]**	***p*** **-Value**	***N***	**Beta** **[95% CI]**	***p*** **-Value**	***N***	**Beta** **[95% CI]**	***p*** **-Value**
ASD mean factor score (standardised)	11,317	−0.11 [−0.15, −0.07]	<0.0005	8744	−0.04 [−0.09, 0.01]	0.092	7500	−0.04 [−0.10, 0.01]	0.143	7114	0.00 [−0.06, 0.06]	0.963

*ASD* autism spectrum disorder, *SCDC* Social and Communication Disorders Checklist, *CCC* Children’s Communication Checklist, *EAS* Emotionality Activity and Sociability

Model 1 = adjusted for sex of the child

Model 2 = adjusted for sex of the child, maternal age, parity, maternal education, social class and financial difficulties

Model 3 = adjusted for sex of the child, maternal age, parity, maternal education, social class, financial difficulties and partner’s smoking

Model 4 = adjusted for sex of the child, maternal age, parity, maternal education, social class, financial difficulties and partner’s smoking and maternal depression Crude model (no sex adjustment)

The same analyses conducted by pregnancy trimester showed similar results, with the strongest associations in the second trimester (Supplemental Tables S2, S3 and S4).

Partner’s smoking associations in sex-adjusted models mirrored those of the mother, except for repetitive behaviour where the association was weaker. There was evidence for confounding. When adjusting for maternal smoking and depression, the association with SCDC score remained, whereas the association with ASD factor mean score was shifted towards the null (Table 3). The protective effect on ASD diagnosis was also weaker after adjusting for socioeconomic covariates and maternal depression.

**Table 3:** Association of partner smoking during pregnancy with child’s autistic spectrum disorder and ASD traits in models without adjustment and with adjustment for socioeconomic status, maternal smoking and maternal depression

Outcome	Model 1			Model 2			Model 3			Model 4		
	*N*	OR [95% CI]	*p*-Value	*N*	OR [95% CI]	*p*-Value	*N*	OR [95% CI]	*p*-Value	*N*	OR [95% CI]	*p*-Value
ASD	10,995	0.51 [0.35, 0.75]	0.001	8307	0.56 [0.35, 0.89]	0.014	7684	0.58 [0.35, 0.96]	0.035	7274	0.60 [0.36, 1.02]	0.057
SCDC score	6879	1.56 [1.30, 1.87]	<0.0005	5784	1.39 [1.13, 1.71]	<0.002	5380	1.32 [1.05, 1.67]	0.018	5135	1.34 [1.06, 1.70]	0.014
CCC coherence (reverted)	6616	1.05 [0.89, 1.24]	0.573	5582	0.99 [0.82, 1.20]	0.926	5182	0.93 [0.74, 1.15]	0.484	4952	0.91 [0.73,1.14]	0.416
Repetitive behaviour	7284	1.11 [0.92, 1.33]	0.270	6087	1.08 [0.87, 1.33]	0.504	5665	1.05 [0.83, 1.34]	0.686	5415	1.05 [0.82, 1.34]	0.720
EAS sociability (reverted)	8421	1.10 [0.96, 1.26]	0.174	6920	1.02 [0.87, 1.20]	0.835	6421	1.09 [0.91, 1.31]	0.333	6119	1.08 [0.90, 1.30]	0.397
	***N***	**Beta [95% CI]**	***p*** **-Value**	***N***	**Beta** **[95% CI]**	***p*** **-Value**	***N***	**Beta** **[95% CI]**	***p*** **-Value**	***N***	**Beta** **[95% CI]**	***p*** **-Value**
ASD mean factor score (standardised)	10,315	−0.09 [−0.13, −0.5]	<0.0005	8098	−0.03 [−0.08, 0.01]	0.126	7500	−0.02 [−0.06, 0.03]	0.526	7114	−0.01 [−0.06, 0.04]	0.769

*ASD* autism spectrum disorder, *SCDC* Social and Communication Disorders Checklist, *CCC* Children’s Communication Checklist, *EAS* Emotionality Activity and Sociability

Model 1 = adjusted for sex of the child

Model 2 = adjusted for sex of the child, maternal age, parity, maternal education, social class and financial difficulties

Model 3 = adjusted for sex of the child, maternal age, parity, maternal education, social class, financial difficulties and maternal smoking

Model 4 = adjusted for sex of the child, maternal age, parity, maternal education, social class, financial difficulties and maternal smoking and maternal depression

Among the five different smoking methylation scores, the Elliott score was explaining 58% of the variance in maternal pregnancy smoking and was taken forward in the analysis (Supplemental Table S5). Using this score, the average smoking methylation score difference between non-smokers and smokers was 9.85 points [95% confidence interval = 9.21–10.49] (*n* = 886, *p* < 0.0005). The methylation score was able to separate smokers and non-smokers according to a threshold of 5.44 points at 73% sensitivity and 96% specificity (Supplemental Figure S1). Due to the reduced sample size in the methylation sub-sample, the analysis on diagnosed ASD was only carried out without adding any covariates to the model. There was no evidence of an association between methylation score and ASD or autistic traits (Table 4).

**Table 4:** Association of maternal smoking during pregnancy as estimated by epigenetic score with child’s autism spectrum disorder and ASD traits in models without adjustment and with adjustment for socioeconomic status, partner’s smoking and maternal depression

Outcome	Model 1			Model 2			Model 3			Model 4		
	*N*	OR [95% CI]	*p*-Value	*N*	OR [95% CI]	*p*-Value	*N*	OR [95% CI]	*p*-Value	*N*	OR [95% CI]	*p*-Value
Any ASD^a^	964	1.07 [0.96, 1.18]	0.245	–	–	–	–	–		–	–	–
SCDC score	877	1.03 [0.98, 1.09]	0.191	763	1.00 [0.94, 1.06]	0.961	653	1.00 [0.93, 1.08]	0.966	640	1.00 [0.93, 1.07]	0.901
CCC coherence (reverted)	879	1.00 [0.95, 1.05]	0.993	766	0.98 [0.93, 1.04]	0.586	656	0.97 [0.91, 1.04]	0.456	644	0.97 [0.90, 1.03]	0.360
Repetitive behaviour	866	0.95 [0.89, 1.01]	0.095	763	0.96 [0.89, 1.03]	0.269	648	0.96 [0.89, 1.04]	0.372	636	0.97 [0.88, 1.04]	0.348
EAS sociability (reverted)	898	0.99 [0.94, 1.04]	0.574	791	0.97 [0.92, 1.03]	0.325	676	0.96 [0.90, 1.03]	0.295	664	0.96 [0.90, 1.03]	0.226
ASD mean factor score (standardised)	963	0.00 [−0.01, 0.01]	0.964	832	0.01 [−0.01, 0.02]	0.371	705	0.01 [0.00, 0.02]	0.145	691	0.01 [0.00, 0.02]	0.100

*ASD* autism spectrum disorder, *SCDC* Social and Communication Disorders Checklist, *CCC* Children’s Communication Checklist, *EAS* Emotionality Activity and Sociability

Model 1 = adjusted for sex of the child

Model 2 = adjusted for sex of the child, maternal age, parity, maternal education, social class and financial difficulties

Model 3 = adjusted for sex of the child, maternal age, parity, maternal education, social class, financial difficulties and partner’s smoking

Model 4 = adjusted for sex of the child, maternal age, parity, maternal education, social class, financial difficulties and partner’s smoking and maternal depression

^a^Crude model (no sex adjustment)

The number of A alleles at the SNP rs1051730:G > A in the *CHRNA3* locus was associated with heaviness of smoking during pregnancy (Supplementary Table S6) at all three trimesters. The effect was more pronounced in the first trimester with each additional A alleles being associated with 1 more cigarette per day on average. Genetic variation at rs1051730:G > A was not associated with any of the potential confounders (Supplemental Table S7). In pregnancy smokers, genetic variation at the *CHRNA3* locus was not associated with any ASD or ASD-related traits, whereas in pregnancy non-smokers each additional A allele was associated with improved CCC speech coherence score (Table 5).

**Table 5:** Effect of additional A alleles at rs1051730 on child’s autism spectrum disorder and ASD traits

	Pregnancy smokers			Pregnancy non-smokers		
Outcome	N	OR [95% CI]	*p*-Value	*N*	OR [95% CI]	*p*-Value
Any ASD	2037	0.79 [0.40, 1.56]	0.504	5399	1.09 [0.77, 1.53]	0.628
SCDC score	1085	0.89 [0.66, 1.20]	0.446	3911	0.95 [0.78, 1.15]	0.575
CCC coherence (reverted)	1002	0.87 [0.64, 1.18]	0.373	3923	0.80 [0.68, 0.95]	0.010
Repetitive behaviour	1197	0.81 [0.59, 1.11]	0.197	4152	0.90 [0.74, 1.09]	0.277
EAS sociability (reverted)	1417	1.07 [0.84, 1.37]	0.572	4659	1.04 [0.91, 1.19]	0.563
	***N***	**Beta [95% CI]**	***p*** **-Value**	***N***	**Beta [95% CI]**	***p*** **-Value**
ASD factor mean score (standardised)	1876	0.03 [−0.03, 0.10]	0.345	5316	0.00 [−0.04, 0.04]	0.998

*ASD* autism spectrum disorder, *SCDC* Social and Communication Disorders Checklist, *CCC* Children’s Communication Checklist, *EAS* Emotionality Activity and Sociability

## Discussion

In this population-based birth cohort study with prospectively collected data, we used a number of analytic strategies to study the association between maternal smoking in pregnancy and autism-related outcomes. We observed some initial evidence of an association between exposure to maternal smoking on behavioural traits linked with ASD, which was attenuated after accounting for potential confounding factors. We found that partner’s smoking is also associated with autistic traits in a similar way to maternal smoking further suggesting that the association is unlikely to be due to an intrauterine effect but that it is more likely to reflect shared confounding. Our analyses using an epigenetic score to reduce the likelihood of reporting bias for maternal smoking status and using a genetic proxy for smoking heaviness in a MR framework also did not support the possibility of a causal association, although both these analyses had lower power to detect an effect.

Our results are in line with the most recent meta-analysis based on 22 studies where the evidence on the association between maternal smoking and ASD has been reviewed and the pooled estimate shows a null association^19^. The studies included in this meta-analysis are very heterogeneous and location of the study, study design, sample size and maternal smoking assessment seem to moderate the association. Particularly, positive associations are more likely to be found in locations outside of Europe and the United States, studies of smaller sample sizes, lower quality, with a case-control design, postnatal assessment of maternal smoking, lack of adjustment for potential confounders and parental reports of ASD diagnosis. These studies might have suffered from selection bias due to small selected samples, recall bias due to postnatal ascertainment and confounding bias. In comparison, our study has strengths in all these areas. Moreover, our study adds to the meta-analysis by showing the absence of evidence for an association with symptoms of autism as measured by the ASD traits.

Interestingly, the estimates from the models not adjusted for socioeconomic confounders showed an effect of maternal smoking in opposite directions, with a reduction in risk of ASD diagnosis and an increase in risk of ASD-related traits. However, the evidence for the reduction in ASD is not strong when considering the number of tests conducted (Bonferroni *p*-value threshold is 0.008 per model tested and six outcomes). If this is a true finding, it might be the reflection of the different socioeconomic patterning of ASD diagnosis and ASD-related traits, with children of higher income families being diagnosed for ASD more often than lower income ones^50^ and children with non-clinical autistic traits in the general population coming from low-income families more than higher-income ones^51^. Since maternal smoking is associated with ASD and autistic traits in opposite directions, this strengthens the idea that maternal smoking is a reflection of socioeconomic status rather than a causal intrauterine factor for autism.

The main strength of our study is the integration of evidence from several different epidemiological approaches that have differing and unrelated sources of bias, to understand if maternal smoking during pregnancy confers a higher risk of developing ASD or ASD-related traits^52^. Our study strengthens the hypothesis of no association by using multivariable regression analysis (where maternal smoking is both self-reported and measured using an epigenetic biomarker), partner’s smoking as a negative control and a MR approach. Other studies have reported a positive association between maternal smoking and children’s behavioural difficulties or neurodevelopment in general (reviewed in ref. ^53^), which seems to support the association reported here in sex-adjusted models on social communication and repetitive behaviours. It has been suggested that the associations found in previous studies are due to unmeasured confounding. In our study we further adjusted for several socioeconomic confounders to reduce this bias and the concordance between the mother’s and partner’s effect estimates leads to shared unmeasured confounding as the most plausible explanation. A similar pattern of matching maternal and paternal effects of smoking on children’s neuro-behavioural outcomes was observed previously on ADHD^54^, suggesting a more general confounding structure that includes other behavioural issues. Although adjusting for partner’s smoking might not provide a point estimate of the causal effect of maternal smoking in the presence of differential measurement error such as reporting bias^55^, when compared with the other approaches the results weaken the hypothesis of a direct intrauterine effect of maternal smoking. Further adjustment for maternal depression additionally attenuated the estimates. This might be due to women with depression being more likely to smoke during pregnancy as smoking rates are higher in persons with mental illness^56^ and to an association of depression in pregnancy with ASD which has been previously reported^21,22^.

The MR analysis minimises the possibility of confounding due to the randomisation of the alleles at conception. Such analysis is possible on cohorts like the ALSPAC where the maternal genotype across the genome has been measured. Smoking behaviour can be instrumented because the genotype at rs1051730:G > A within the *CHRNA3* gene is strongly associated with smoking heaviness^25,57–59^ and the association has been replicated in this study. The only association between this genetic variant and the autistic-related outcomes in this study is within the pregnancy non-smokers, where an effect of smoking heaviness is not expected. The evidence for this association is weak considering the number of tests carried out (above the Bonferroni *p*-value threshold for six outcomes investigated of 0.05/6 = 0.008). If this effect is true, this could be due to pleiotropy, i.e. the genetic variant affecting the outcome via pathways other than smoking. A look-up analysis performed on MR-Base (www.mrbase.org; last access on 07/02/2018) found an association of rs1051730:G > A with several smoking variables and with schizophrenia at Bonferroni-corrected threshold *p*-value = 3.08 × 10^−5^ (Supplementary Table S8). The pragmatic language coherence score that we identified being affected by this genetic variant in non-smokers could therefore be the result of a direct effect of maternal *CHRNA3* on language development bypassing smoking, for example, via the mother’s own language development.

Another advantage of using a prospective cohort is that it minimises the possibility of recall bias since all the parents were asked about their smoking habits during pregnancy. To further reduce measurement bias we utilised an epigenetic score calculated from the maternal blood DNA methylation profiles. The score did not give a perfect distinction between smokers and non-smokers either due to reporting bias from self-report or measurement error and it added an extra approach to examine the association of smoking behaviour during pregnancy on neurodevelopment. In this analysis, the fact that we did not find the same associations of maternal smoking with social communication and repetitive behaviour could be due to the relatively small sample size, which did not allow as much power as for the main analysis.

The main limitation of this study is the low number of ASD cases in the study population and the small sample size in the genetic and epigenetic analysis, which may have compromised the statistical power for comparisons. Moreover, since ASD ascertainment included reported or possible ASD diagnosis, ASD cases are likely to include false positives. However, the results are consistent across the different analyses suggesting that a causative link between maternal smoking during pregnancy and autistic features is unlikely.

In conclusion, taken together our study did not find evidence to support a causal association between maternal smoking during pregnancy and ASD or autistic-like behaviours via intrauterine mechanisms.

## Electronic supplementary material

Supplemental Material
