# Supplementary material for: Maternal smoking during pregnancy and autism: using causal inference methods in a birth cohort study
Source: Transl Psychiatry. 2018 Nov 29;8:262. doi: 10.1038/s41398-018-0313-5 (PMC6265272; doi:10.1038/s41398-018-0313-5)
Supplement: Supplementary file 1 — Supplemental Material [file 41398_2018_313_MOESM1_ESM.docx]

**SUPPLEMENTAL METHODS**

**Epigenetic data**

The DNA methylation data used to create the epigenetic score were generated from as part of the Accessible Resource for Integrated Epigenomic Studies (ARIES) ^1^. Briefly, DNA was extracted from whole blood collected from the mothers during pregnancy and cord blood was collected at birth. The DNA samples were bisulphite converted using the Zymo EZ DNA methylationTM kit (Zymo, Irvine, CA) and DNA methylation was measured using the Illumina Infinium HumanMethylation450 BeadChip and Illumina iScan. For each sample the proportion of methylated DNA molecules at each CpG site is represented as beta=M/(M+U+100), where M and U denote methylated and unmethylated signal intensities, respectively. Quality control steps and normalization procedures were run using meffil R package (available at <https://github.com/perishky/meffil>).

**Genetic data**

The genetic data used in the Mendelian randomization analysis were extracted from the ALSPAC genetic database**.** This was generated using the Illumina human660W-quad and the IlluminaGenomeStudio calling algorithm. Quality Control measures included the removal of SNPs with more than 5% of missingness, a Hardy-Weinberg-Equilibrium P-value lower than 10^-6^ and a minor allele frequency of less than 1%. Samples with more than 5% missingness, indeterminate X chromosome heterozygosity or extreme autosomal heterozygosity were excluded. SNP imputation was carried out against the 1000 Genome Project database ([www.1000genomes.org](http://www.1000genomes.org)) ^2^.

**SUPPLEMENTAL TABLES**

**Table S1.** Association of covariates with mother’s and partner’s smoking status during pregnancy.

|  |  | **Mother smoked during pregnancy** | | | **Partner smoked during pregnancy** | | |
| --- | --- | --- | --- | --- | --- | --- | --- |
|  |  | **No** | **Yes** | **p-value ^1^** | **No** | **Yes** | **p-value^1^** |
| Sex | Male (%) | 51.03 | 53.63 |  | 51.17 | 52.22 |  |
|  | Female (%) | 48.97 | 46.37 | 0.009 | 48.83 | 47.78 | 0.330 |
| Social class | Non-manual (%) | 84.64 | 68.28 |  | 84.58 | 73.54 |  |
| Maternal education | Manual (%) | 15.36 | 31.72 | <0.0005 | 15.42 | 26.46 | <0.0005 |
|  | No university degree (%) | 83.35 | 95.59 |  | 82.15 | 92.85 |  |
|  | University degree (%) | 16.65 | 4.41 | <0.0005 | 17.85 | 7.15 | <0.0005 |
| Parity | 0 (%) | 43.45 | 44.58 |  | 46.81 | 45.90 |  |
|  | ≥1 (%) | 56.55 | 55.42 | 0.259 | 53.19 | 54.10 | 0.400 |
| Financial difficulties | Score <9 (%) | 93.14 | 82.29 |  | 93.85 | 86.56 |  |
|  | Score ≥9 (%) | 6.86 | 17.71 | <0.0005 | 6.15 | 13.44 | <0.0005 |
| Maternal age (years) | Mean (sd) | 29.07 (0.05) | 26.29 (0.08) | <0.0005 | 28.90 (0.06) | 27.03 (0.09) | <0.0005 |
| EPDS score | Mean (sd) | 6.25 (0.05) | 8.51 (0.09) | <0.0005 | 6.39 (0.06) | 7.48 (0.09) | <0.0005 |
|  |  |  |  |  |  |  |  |

^1^ from chi-square test (categorical variables) or from t-test (continuous variables)

EPDS= Edinburgh Postnatal Depression Scale

**Table S2.** Association of maternal smoking during pregnancy with child’s autism spectrum disorder and ASD traits without adjustment and with adjustment for socio-economic status, partner’s smoking and maternal depression in the first pregnancy trimester.

| **Outcome** | **Model 1** | | | **Model 2** | | | **Model 3** | | | **Model 4** | | |  |
| --- | --- | --- | --- | --- | --- | --- | --- | --- | --- | --- | --- | --- | --- |
|  | **N** | **OR**  **[95% C.I.]** | **p-value** | **N** | **OR**  **[95% C.I.]** | **p-value** | **N** | **OR**  **[95% C.I.]** | **p-value** | **N** | **OR**  **[95% C.I.]** | **p-value** |  |
| ASD | 13082 | 0.62  [0.40, 0.94] | 0.026 | 9,654 | 0.64  [0.37, 1.01] | 0.103 | 8,280 | 0.76  [0.40, 1.41] | 0.381 | 9,090 | 0.68  [0.38, 1.20] | 0.184 |  |
| SCDC score | 7924 | 1.55  [1.28, 1.88] | <0.0005 | 6567 | 1.49  [1.19, 1.86] | <0.001 | 5768 | 1.29  [0.99, 1.67] | 0.056 | 6246 | 1.40  [1.11, 1.76] | 0.005 |  |
| CCC coherence  (reverted) | 7608 | 1.17  [0.97, 1.40] | 0.104 | 6315 | 1.06  [0.85, 1.33] | 0.604 | 5566 | 1.10  [0.86, 1.42] | 0.441 | 6019 | 1.01  [0.80, 1.28] | 0.923 |  |
| Repetitive  behaviour | 8405 | 1.25  [1.02, 1.52] | 0.031 | 6916 | 1.18  [0.93, 1.51] | 0.170 | 6069 | 1.10  [0.84, 1.45] | 0.480 | 6580 | 1.07  [0.83, 1.37] | 0.607 |  |
| EAS sociability  (reverted) | 9812 | 0.97  [0.83, 1.13] | 0.681 | 7923 | 0.87  [0.72, 1.06] | 0.169 | 6899 | 0.90  [0.73, 1.15] | 0.340 | 7520 | 0.84  [0.69, 1.03] | 0.091 |  |
|  |  |  |  |  |  |  |  |  |  |  |  |  |  |
|  | **N** | **Beta**  **[95% C.I.]** | **p-value** | **N** | **Beta**  **[95% C.I.]** | **p-value** | **N** | **Beta**  **[95% C.I.]** | **p-value** | **N** | **Beta**  **[95% C.I.]** | **p-value** |  |
| ASD  mean  factor score  (standardized) | 12180 | -0.10  [-0.14, -0.06] | <0.0005 | 9384 | -0.05  [-0.10, 0.002] | 0.064 | 8074 | -0.04  [-0.10, 0.00] | 0.143 | 8858 | 0.00  [-0.06, 0.04] | 0.706 |  |

**Table S3.** Association of maternal smoking during pregnancy with child’s autism spectrum disorder and ASD traits without adjustment and with adjustment for socio-economic status, partner’s smoking and maternal depression in the second pregnancy trimester.

| **Outcome** | **Model 1** | | | **Model 2** | | | **Model 3** | | | **Model 4** | | |  |
| --- | --- | --- | --- | --- | --- | --- | --- | --- | --- | --- | --- | --- | --- |
|  | **N** | **OR**  **[95% C.I.]** | **p-value** | **N** | **OR**  **[95% C.I.]** | **p-value** | **N** | **OR**  **[95% C.I.]** | **p-value** | **N** | **OR**  **[95% C.I.]** | **p-value** |  |
| ASD | 13094 | 0.61  [0.38, 0.97] | 0.038 | 9665 | 0.83  [0.47, 1.44] | 0.500 | 8290 | 1.07  [0.57, 2.02] | 0.823 | 9101 | 0.87  [0.49, 1.56] | 0.650 |  |
| SCDC score | 7927 | 1.70  [1.39, 2.08] | <0.0005 | 6572 | 1.62  [1.27, 2.06] | <0.001 | 5772 | 1.49  [1.14, 1.96] | 0.004 | 6252 | 1.54  [1.20, 1.97] | 0.005 |  |
| CCC coherence  (reverted) | 7610 | 1.35  [1.11, 1.64] | 0.003 | 6320 | 1.24  [0.98, 1.57] | 0.076 | 5570 | 1.32  [1.01, 1.72] | 0.045 | 6025 | 1.15  [0.90, 1.48] | 0.254 |  |
| Repetitive  behaviour | 8409 | 1.31  [1.06, 1.62] | 0.014 | 6922 | 1.36  [1.05, 1.75] | 0.019 | 6074 | 1.30  [0.97, 1.73] | 0.074 | 6587 | 1.23  [0.95, 1.61] | 0.121 |  |
| EAS sociability  (reverted) | 9817 | 1.07  [0.91, 1.26] | 0.417 | 7931 | 0.93  [0.76, 1.15] | 0.514 | 6906 | 0.96  [0.77, 1.12] | 0.752 | 7529 | 0.87  [0.70, 1.08] | 0.201 |  |
|  |  |  |  |  |  |  |  |  |  |  |  |  |  |
|  | **N** | **Beta**  **[95% C.I.]** | **p-value** | **N** | **Beta**  **[95% C.I.]** | **p-value** | **N** | **Beta**  **[95% C.I.]** | **p-value** | **N** | **Beta**  **[95% C.I.]** | **p-value** |  |
| ASD  mean  factor score  (standardized) | 12189 | -0.14  [-0.18, -0.09] | <0.0005 | 9393 | -0.07  [-0.13, -0.02] | 0.005 | 8082 | -0.07  [-0.14, -0.01] | 0.014 | 8867 | -0.04  [-0.10, 0.01] | 0.121 |  |

**Table S4.** Association of maternal smoking during pregnancy with child’s autism spectrum disorder and ASD traits without adjustment and with adjustment for socio-economic status, partner’s smoking and maternal depression in the third pregnancy trimester.

| **Outcome** | **Model 1** | | | **Model 2** | | | **Model 3** | | | **Model 4** | | |  |
| --- | --- | --- | --- | --- | --- | --- | --- | --- | --- | --- | --- | --- | --- |
|  | **N** | **OR**  **[95% C.I.]** | **p-value** | **N** | **OR**  **[95% C.I.]** | **p-value** | **N** | **OR**  **[95% C.I.]** | **p-value** | **N** | **OR**  **[95% C.I.]** | **p-value** |  |
| ASD | 12632 | 0.71  [0.46, 1.09] | 0.120 | 9669 | 0.71  [0.41, 1.25] | 0.239 | 8293 | 1.00  [0.54, 1.85] | 0.993 | 9105 | 0.77  [0.43, 1.37] | 0.371 |  |
| SCDC score | 7902 | 1.56  [1.27, 1.92] | <0.0005 | 6576 | 1.50  [1.19, 1.90] | 0.001 | 5776 | 135  [1.03, 1.76] | 0.027 | 6255 | 1.43  [1.12, 1.82] | 0.004 |  |
| CCC coherence  (reverted) | 7592 | 1.36  [1.12, 1.65] | 0.002 | 6324 | 1.22  [0.97, 1.53] | 0.095 | 5574 | 1.28  [0.99, 1.66] | 0.060 | 6028 | 1.14  [0.90, 1.45] | 0.276 |  |
| Repetitive  behaviour | 8379 | 1.29  [1.04, 1.59] | 0.020 | 6927 | 1.27  [0.99, 1.63] | 0.062 | 6078 | 1.25  [0.94, 1.65] | 0.121 | 6591 | 1.16  [0.89, 1.51] | 0.262 |  |
| EAS sociability  (reverted) | 9775 | 1.04  [0.89, 1.22] | 0.631 | 7936 | 0.89  [0.73, 1.09] | 0.254 | 6910 | 0.91  [0.73, 1.14] | 0.410 | 7533 | 0.84  [0.68, 1.03] | 0.096 |  |
|  |  |  |  |  |  |  |  |  |  |  |  |  |  |
|  | **N** | **Beta**  **[95% C.I.]** | **p-value** | **N** | **Beta**  **[95% C.I.]** | **p-value** | **N** | **Beta**  **[95% C.I.]** | **p-value** | **N** | **Beta**  **[95% C.I.]** | **p-value** |  |
| ASD  mean  factor score  (standardized) | 12065 | -0.14  [-0.18, -0.09] | <0.0005 | 9398 | -0.07  [-0.12, -0.01] | 0.005 | 8086 | -0.08  [-0.14, -0.02] | 0.009 | 8872 | -0.03  [-0.09, 0.02] | 0.216 |  |

**Table S5.** Proportion of variance in pregnancy smoking explained by smoking methylation scores.

| **Score** | **Pseudo-R^2^** |
| --- | --- |
| Elliott | 0.58 |
| Joehanes | 0.41 |
| Joehanes-PC-adjusted | 0.44 |
| Joubert | 0.19 |
| Joubert-PC-adjusted | 0.23 |

**Table S6.** Effect of additional A alleles at *CHRNA3* rs1051730 on heaviness of smoking (cigarettes per day) in pregnancy smokers by trimester.

|  |  | **Beta** | **95% C.I.** | **p-value** |
| --- | --- | --- | --- | --- |
| *CHRNA3* | First trimester (N=1959) | 0.99 | 0.48, 1.49 | <0.0005 |
|  | Second trimester (N=1969) | 0.67 | 0.14, 1.20 | 0.014 |
|  | Third trimester (N=1764) | 0.56 | 0.05, 1.07 | 0.031 |

**Table S7**. Effect of rs1051730 A alleles on potential confounders.

|  |  | **0** | **1** | **2** | **p-value^1^** |
| --- | --- | --- | --- | --- | --- |
| Sex | Male (N) | 1979 | 1922 | 502 |  |
|  | Female (N) | 1949 | 1936 | 985 | 0.776 |
| Social class | Non-manual (N) | 2399 | 2357 | 608 |  |
| Maternal education | Manual (N) | 520 | 517 | 144 | 0.694 |
|  | No university degree (N) | 3009 | 2972 | 772 |  |
|  | University degree (N) | 488 | 479 | 114 | 0.692 |
| Parity | 0 (N) | 1665 | 1625 | 412 |  |
|  | ≥1 (N) | 1944 | 1921 | 494 | 0.927 |
| Financial difficulties | Score <9 (N) | 3115 | 3054 | 787 |  |
|  | Score ≥9 (N) | 302 | 302 | 80 | 0.931 |
| Maternal age (years) | Mean (sd) | 28.30 (4.76) | 28.38 (4.77) | 28.37 (4.76) | 0.653 |
| EPDS score | Mean (sd) | 6.68 (4.82) | 6.87 (4.77) | 6.55 (5.04) | 0.415 |
|  |  |  |  |  |  |

^1^ from χ^2^or ANOVA tests (categorical or continuous, respectively)

**Table S8.** Associations of rs1051730 at Bonferroni p-value<0.05 from hypothesis-free look-up analysis in MR-Base.

| **Trait** | **Beta^1^** | **SE** | **N total** | **N cases** | **N controls** | **Effect allele** |
| --- | --- | --- | --- | --- | --- | --- |
| Pack years adult smoking as proportion of life span exposed to smoking | 0.085452 | 0.004632 | 101726 | NA | NA | A |
| Number of cigarettes previously smoked daily | 0.084176 | 0.004582 | 78291 | NA | NA | A |
| Pack years of smoking PREVIEW ONLY | 0.078802 | 0.004618 | 101726 | NA | NA | A |
| Lung cancer | 0.29992 | 0.024781 | 27209 | 11348 | 15861 | A |
| Cigarettes smoked per day | 1.0207 | 0.0855 | 68028 | NA | NA | A |
| Light smokers at least 100 smokes in lifetime | -0.02791 | 0.00249 | 90517 | NA | NA | A |
| Number of cigarettes currently smoked daily (current cigarette smokers) | 0.077392 | 0.007044 | 23205 | NA | NA | A |
| Difficulty not smoking for 1 day | 0.090107 | 0.008882 | 23332 | NA | NA | A |
| Lung adenocarcinoma | 0.292583 | 0.038014 | 18336 | 3442 | 14894 | A |
| Squamous cell lung cancer | 0.279984 | 0.037492 | 18313 | 3275 | 15038 | A |
| Time from waking to first cigarette | -0.09333 | 0.011366 | 23265 | NA | NA | A |
| Forced expiratory volume in 1-second (FEV1) predicted percentage | -0.03648 | 0.004512 | 110423 | NA | NA | A |
| Illnesses of father: Lung cancer | 0.005644 | 0.000782 | 292053 | NA | NA | A |
| Forced expiratory volume in 1-second (FEV1) Best measure | -0.01617 | 0.002431 | 255492 | NA | NA | A |
| Father's age at death | -0.01984 | 0.002983 | 248726 | NA | NA | A |
| Illnesses of father: Chronic bronchitis/emphysema | 0.005574 | 0.000858 | 292053 | NA | NA | A |
| Forced expiratory volume in 1-second (FEV1) | -0.0137 | 0.002211 | 307638 | NA | NA | A |
| Father still alive | -0.00671 | 0.001105 | 328694 | NA | NA | A |
| Illnesses of mother: Chronic bronchitis/emphysema | 0.00363 | 0.00062 | 308780 | NA | NA | A |
| Father's age at death | 0.026929 | 0.00486 | 75244 | NA | NA | G |
| Number of unsuccessful stop-smoking attempts | 0.022757 | 0.004302 | 75135 | NA | NA | A |
| Forced vital capacity (FVC) Best measure | -0.01159 | 0.002302 | 255492 | NA | NA | A |
| Schizophrenia | 0.056702 | 0.0113 | 82315 | 35476 | 46839 | A |
| Forced vital capacity (FVC) | -0.01004 | 0.002096 | 307638 | NA | NA | A |
| Ever smoked | -0.00585 | 0.001263 | 336067 | NA | NA | A |
| Ever stopped smoking for 6+ months | -0.01175 | 0.002595 | 81538 | NA | NA | A |
| Former vs current smoker | -0.0741 | 0.0169 | 70675 | 23554 | 18415 | A |
| Smoking status: Previous | -0.00535 | 0.001235 | 336024 | NA | NA | A |
| Mouth/teeth dental problems: Dentures | 0.004028 | 0.00097 | 336138 | NA | NA | A |

^1^ Logodds for case/control studies

**Figure S1.** Smoking methylation score in the pregnant smokers and non-smokers.


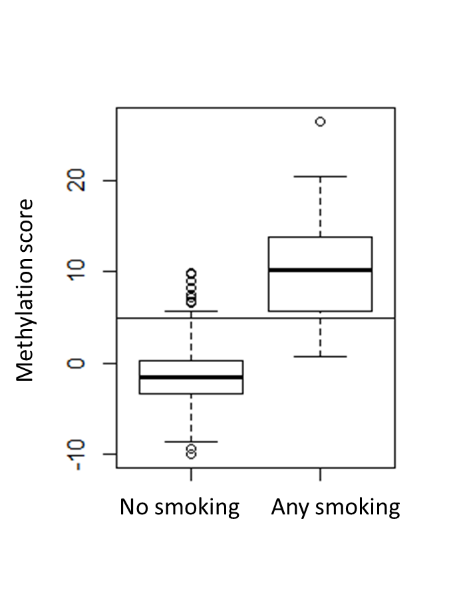


**REFERENCES**

1. Relton CL, Gaunt T, McArdle W, Ho K, Duggirala A, Shihab H*, et al*. Data Resource Profile: Accessible Resource for Integrated Epigenomic Studies (ARIES). *International journal of epidemiology* 2015; **44:** 1181-1190.

2. Genomes Project C, Abecasis GR, Auton A, Brooks LD, DePristo MA, Durbin RM*, et al*. An integrated map of genetic variation from 1,092 human genomes. *Nature* 2012; **491:** 56-65.
